# Genome-Wide Discovery of SSR Markers Based on Whole-Genome Resequencing Data of *Dendrobium officinale*

**DOI:** 10.3390/plants14233589

**Published:** 2025-11-25

**Authors:** Mingmin Zheng, Hang Mi, Pingrong Zhou, Ting Li, Yelin Wang, Jian Liu, Wei Jiang

**Affiliations:** 1 Sichuan Provincial Key Laboratory for Development and Utilization of Characteristic Horticultural Biological Resources, Chengdu Normal University, Chengdu 611130, China; 2 College of Chemistry and Life Sciences, Chengdu Normal University, Chengdu 611130, China; 3 Maize Research Institute, Sichuan Agricultural University, Chengdu 611130, China

**Keywords:** whole-genome resequencing (WGRS), simple sequence repeat (SSR), *Dendrobium officinale*, polymorphism, molecular marker

## Abstract

*Dendrobium officinale*, a perennial epiphytic herb of the orchid family renowned for its ornamental value and diverse medicinal properties, has attracted considerable global attention due to its rarity and increasing market demand. However, large-scale cultivation has led to confusion regarding its germplasm resources and genetic backgrounds, posing significant challenges for the effective conservation, management, and utilization of *D. officinale* germplasm. In this study, we systematically analyzed the abundance and characteristics of simple sequence repeats (SSRs) and developed highly polymorphic genomic SSR markers using whole-genome resequencing (WGRS) data from 15 *D. officinale* genotypes. A total of 494,783 SSRs were identified in the “Niu2020” reference genome. Mononucleotide repeats were the most abundant motifs, followed by dinucleotide repeats, with A/T and AT/TA being the predominant types, respectively. Of the SSRs with unique flanking sequences (unique SSRs), 121,544 out of 388,553 (31.28%) were polymorphic across the 15 genotypes. The polymorphism information content (PIC) values of these SSRs ranged from 0.13 to 0.92, with an average of 0.59. Subsequently, 10,364 pairs of SSR primers were successfully designed from polymorphic SSR loci with PIC values ≥ 0.5 and major allele size differences ≥ 3 bp. Ultimately, 20 primer pairs were randomly selected for experimental validation. Of these, 18 successfully amplified the target fragments and exhibited high levels of polymorphism (PIC values ranged from 0.31 to 0.74), confirming the effectiveness and practical utility of the newly developed markers. The SSR fingerprints and polymorphic markers developed in this study provide a valuable resource and establish a robust technical foundation for enhancing the efficiency of cultivar identification, genetic research, and molecular breeding in *D. officinale*.

## 1. Introduction

*Dendrobium officinale* Kimura et Migo, commonly known as Tiepi Shihu in Chinese, belongs to the genus *Dendrobium*, which is one of the largest genera in the Orchidaceae family. *Dendrobium* contains more than 1500 species, which are widely distributed across the tropical and subtropical regions of Asia and Oceania, with over 80 species reported in China [1,2]. *Dendrobium* species have been used by traditional Chinese medical practitioners to treat diabetes, obesity, hepatitis, rheumatoid arthritis, and various other ailments, owing to their diverse pharmacological properties [3,4]. Due to its high content of bioactive compounds, *D. officinale*, often referred to as “the first of the nine Chinese fairy herbs”, is one of the five *Dendrobium* species listed in the 2020 edition of the Chinese Pharmacopoeia. Among *Dendrobium* herbs in China, *D. officinale* is generally considered by traditional Chinese medical practitioners to be the most therapeutically effective [3]. As early as 1,300 years ago, it was documented as “one of the most famous and precious *Dendrobium* species” [5]. *D. officinale* is predominantly outcrossing and exhibits self-incompatibility, whereby cross-pollination results in a substantially higher fruit set rate [6]. This reproductive characteristic contributes to its high genetic variability and complex population structure. This species is diploid, with a chromosome number of 2n = 2x = 38 and an estimated genome size of 1.23 Gb [7]. The stems and leaves of *D. officinale* contain a variety of bioactive compounds, including polysaccharides, polyphenols, flavonoids, alkaloids, and amino acids, among which polysaccharides are regarded as the primary active constituents [1,8,9,10,11,12]. Several in vitro and in vivo studies have demonstrated the diverse pharmacological activities of *D. officinale*, including antitumor, antidiabetic, gastroprotective, hepatoprotective, anti-aging, and neuroprotective effects [13,14,15,16,17,18,19]. In addition, *D. officinale* is cultivated as an ornamental plant for its beautiful flowers and distinctive architecture [20]. Consequently, it has become the most popular *Dendrobium* species and is in high demand in China. Unfortunately, wild *D. officinale* has been on the brink of extinction since the 1950s, primarily due to overexploitation and habitat degradation [21]. The IUCN Red List of Threatened Species classifies *D. officinale* as Critically Endangered (CR) (http://www.iucnredlist.org/, accessed on 27 May 2025). Currently, over 90% of *D. officinale* is cultivated using artificial substrates to meet the high demand in China’s healthcare market [22].

Authentication and differentiation of *Dendrobium* species are notoriously difficult because of their morphological resemblance and the complex and overlapping morphological variations both within and among species [23,24,25,26]. For effective conservation, characterization, and utilization of *D. officinale* resources, it is critical to understand genetic variation and evolutionary processes within and among populations. Various molecular markers, such as random amplified polymorphic DNA (RAPD) [27,28,29], amplified fragment length polymorphism (AFLP) [30,31], simple sequence repeat (SSR) [32,33], expressed sequence tag-SSR (EST-SSR) [34,35], inter-SSR (ISSR) [28,31,36,37], and single nucleotide polymorphism (SNP) [38], have been utilized to assess the genetic diversity of *D. officinale*. Despite these achievements, molecular research on *D. officinale* still lags behind that in many crops, primarily due to the limited number of available molecular markers. SSR markers are regarded as among the most efficient and versatile molecular markers due to several desirable genetic attributes, including co-dominant inheritance, reproducibility, high polymorphism, multiallelic nature, extensive genome coverage, and technical simplicity [39,40,41]. These markers have been widely developed and applied in plants for genetic diversity analysis, evolutionary studies, genetic fingerprinting, linkage map construction, quantitative trait loci (QTL) mapping, and molecular marker-assisted breeding [42]. Although hundreds of SSR markers have already been developed for *D. officinale* through various strategies [21,43,44,45,46,47,48], their number, genomic coverage, and polymorphism remain insufficient to meet the needs of advanced genetic research such as high-density genetic map construction, genome comparative mapping, map-based gene cloning, and marker-assisted breeding. The breeding of *D. officinale* is progressively aligning with approaches used in field crops, especially in the transition from traditional selection breeding to hybrid breeding. This trend underscores the growing importance of large-scale molecular marker development, particularly for gene mapping, cloning, and marker-assisted selection.

Compared with traditional approaches, next-generation sequencing (NGS) technology offers a rapid, efficient, and cost-effective strategy for genome-wide molecular marker discovery and development [42,49]. Furthermore, the availability of a high-quality chromosome-level reference genome of *D. officinale* [7] has paved the way for large-scale discovery of molecular markers. In this study, we performed whole-genome resequencing (WGRS) on 14 *D. officinale* genotypes using the Illumina platform. Based on WGRS data from these 14 genotypes and the published reference genome [7], we characterized the abundance and distribution of SSRs, analyzed genome-wide SSR polymorphisms, and developed a set of high-density polymorphic SSR markers. We further conducted experimental validation to evaluate the practical utility of the identified SSR markers. By establishing a high-density genomic SSR marker database, this study provides a valuable resource and an effective tool for cultivar identification, genetic research, and molecular breeding in *D. officinale*.

## 2. Results

### 2.1. Genome-Wide Identification and Characterization of SSRs in D. officinale

According to the search criteria, we identified 494,783 candidate SSRs with mono- to hexa-nucleotide motifs in the *D. officinale* “Niu2020” genome (Table S1), corresponding to an overall density of 430.50 SSRs/Mb (one SSR every 2.32 kb). We further examined the distribution of SSRs with respect to the number of repeat units. The mono- and di-nucleotide repeats were the most abundant types, accounting for 66.57% (329,390) and 18.50% (91,524) of the total SSRs, respectively (Table 1). The number of SSR loci gradually decreased as the motif length increased. For mononucleotide repeats, A/T motifs were predominant (91.72%), whereas AT/TA was the most frequent type among dinucleotide repeats (50.57%) (Table S2). The predominant repeat motifs exhibited a high A/T content, which is consistent with observations in most plant species. Using the e-PCR algorithm, 388,553 SSRs (78.53% of the total) were mapped to unique genomic regions (Table 1). The unique SSRs were unevenly distributed across different genomic regions. The density of unique SSRs was highest in the TES_down_1kb region, followed by the 5’UTR, TSS_up_2kb, 3’UTR, introns, intergenic regions, and finally the CDS region (Table 2).

### 2.2. Identification of Unique SSRs with Polymorphisms

Whole-genome resequencing (WGRS) was performed on 14 *D. officinale* genotypes, generating a total of 673,081,491 reads. The sequencing depth ranged from 8.69× to 12.70×, with an average of 11.00× (Table S3). The 388,553 unique SSR loci were analyzed in each of the 14 genotypes. The average number of unique SSRs across the 14 genomes was 176,015, representing approximately 45.30% of the unique SSR loci identified in the reference genome. Among the 14 genotypes, A34 harbored the highest number of unique SSR loci (204,643), accounting for 52.67% of all unique SSRs. In contrast, Hw3 contained the fewest (139,892), accounting for 36.00% of all unique SSRs (Table S4). Of the 388,553 unique SSRs in the reference genome, 128,841 loci (33.16%) were shared by ten or more of the analyzed genomes and were thus defined as common loci. Among these common SSRs, 121,544 (94.34%) were polymorphic. The average density of polymorphic SSRs was 105.75 SSRs/Mb, with the highest density on chromosome 6 (131.81 SSRs/Mb, or one SSR every 7.59 kb) and the lowest on chromosome 10 (79.42 SSRs/Mb, or one SSR every 12.59 kb) (Table S5).

### 2.3. Analysis of the Frequency and Distribution of Polymorphic SSRs

**Figure 1:**
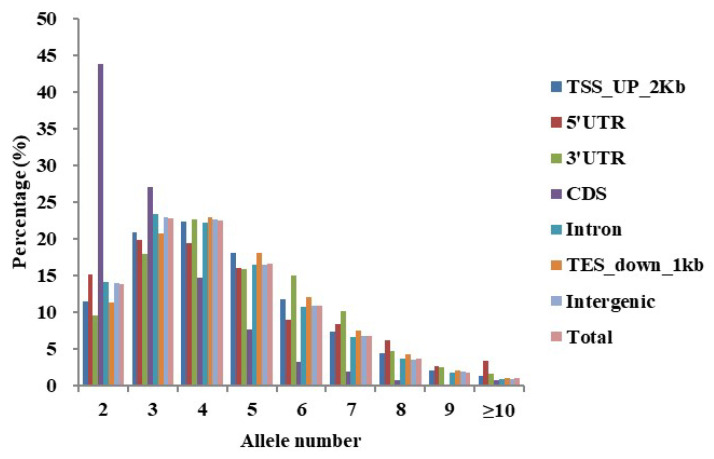
Distribution of SSR allele numbers across genomic regions.

An *in silico* analysis was conducted to compare the number of alleles and PIC values of SSRs (Table S6). The PIC values of polymorphic SSRs ranged from 0.13 to 0.92, with an average of 0.59. A total of 89,835 SSRs exhibited a high level of polymorphism (PIC values ≥ 0.5), accounting for 73.91% of all polymorphic SSRs. The number of alleles per polymorphic locus ranged from 2 to 14, with a mean of 4.16. SSRs with two, three, four, and five alleles were predominant, accounting for 15.83%, 24.70%, 23.40%, and 16.38% of the polymorphic SSRs, respectively. The relative abundance of polymorphic SSRs categorized by allele number was generally consistent across most genomic regions, with the exception of the CDS region. In the CDS region, di-allelic SSRs accounted for a notably higher proportion (43.81%) than in other genomic regions. Correspondingly, the proportion of SSRs harboring four or more alleles was lower in the CDS region compared to other genomic regions (Figure 1).

### 2.4. Development of SSR Markers

Unique SSR loci with PIC values ≥ 0.5 and major allele size differences ≥ 3 bp were selected from the “Niu2020” reference genome for primer design. Primers targeting SSR loci were designed to amplify PCR products ranging from 70 to 300 bp, which can be efficiently resolved on a polyacrylamide gel. Ultimately, 10,364 primer pairs were designed from the flanking regions of these loci. Table S7 details the exact positions of these SSRs in the “Niu2020” genome, along with comprehensive information including repeat motifs, primer sequences, amplicon sizes, allele numbers, PIC values, major allele size differences, as well as e-PCR product counts across the *D. officinale* genomes. These data provide a valuable resource for selecting primers in practical applications.

### 2.5. Validation of SSR Markers for Amplification Efficiency and Polymorphism

**Figure 2:**
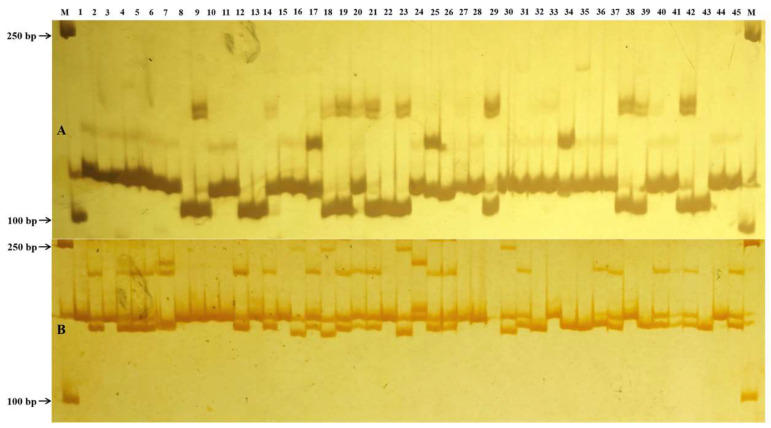
Validation of SSR polymorphisms in 45 *Dendrobium officinale* germplasm accessions. Polyacrylamide gel electrophoresis profiles of microsatellite alleles amplified with Chr3-3538709 (**A**) and Chr4-23399662 (**B**). Lane M, DL2000 DNA marker; Lanes 1–45, PCR products from the corresponding accessions listed in Table S8.

To validate the *in silico* findings, 20 SSR markers with mono- to penta-nucleotide motifs were randomly selected for PCR amplification using genomic DNA from 45 *D. officinale* germplasm accessions of diverse geographic origins. In the 15 sequenced *D. officinale* genotypes, the number of alleles per locus ranged from 2 to 5, with an average of 3.20, while the PIC values ranged from 0.50 to 0.71, with an average of 0.58 (Table 3). Among the primer sets tested, 19 SSR markers produced specific amplification products, yielding clear bands of the expected sizes. Eighteen of these markers exhibited high allelic diversity and good transferability in the tested germplasm accessions (Figure 2). However, one SSR marker produced only a single amplification band. These 18 polymorphic SSR loci yielded a total of 67 alleles. The number of alleles per locus ranged from 2 to 6, with an average of 3.72, while the PIC values ranged from 0.31 to 0.74, with an average of 0.56 (Table 3). Furthermore, nine SSR markers yielded alleles consistent with the *in silico* predictions, while six produced more and three fewer alleles during experimental validation. Interestingly, heterozygous bands were detected in PCR products amplified by several primers, suggesting a complex genetic background in the tested germplasm accessions. Overall, these results indicate that the newly developed genomic-SSR markers are informative and valuable, with most markers demonstrating reliable amplification and polymorphism.

## 3. Discussion

SSRs, consisting of tandem repeats of one to six nucleotides, are widely distributed across plant genomes. A comprehensive survey of the high-quality, chromosome-level reference genome of *D. officinale* “Niu2020” identified 494,783 candidate SSRs, with an average density of one SSR every 2.32 kb. This density reflected a relatively high SSR abundance in *D. officinale*, exceeding that reported for rice [50] (one SSR every 3.6 kb) and maize [51] (one SSR every 7.93 kb). SSR frequency can be influenced by search criteria, dataset size, identification tools, and the species analyzed [52]. SSR characterization revealed that mononucleotide repeats were the most abundant, followed by dinucleotide repeats, whereas tetra-, penta-, and hexa-nucleotide motifs were relatively rare, together accounting for less than 10% of all SSRs. This distribution pattern is consistent with previous observations in maize and rice. Overall, 388,553 SSRs (78.53%) possessed unique flanking sequences, highlighting their high potential as genetic markers. These unique SSRs were unevenly distributed across genomic regions, with the SSR density in CDS regions significantly lower than in other regions.

Conventional methods for developing SSR markers, such as screening genomic DNA libraries or microsatellite-enriched libraries, were labor-intensive, costly, and time-consuming [42,53], and yielded only a limited number of usable markers. The advent of next-generation sequencing (NGS) technologies has markedly accelerated the development of large-scale SSR markers by improving efficiency, accuracy, and throughput [42,49]. Prior to the availability of genomic sequence data for *D. officinale*, expressed sequence tag (EST) databases served as a resource for developing genic SSR markers. For instance, Lu et al. developed 110 novel *D. officinale* EST-SSRs by screening a cDNA library [45], while Xu et al. designed 68 EST-SSRs using transcriptome data from two cultivated varieties [47]. Compared with genic SSR markers, genomic SSR markers display higher polymorphism and broader genome coverage, rendering them more effective for distinguishing closely related genotypes [41,52]. Therefore, genomic SSR markers are generally considered superior for fingerprinting and variety identification [41,52].

We adopted a novel approach proposed by Liu et al. [54] to discover polymorphic SSR markers. This method involves aligning and comparing the genome sequences of multiple germplasm accessions against the reference genome to detect and genotype variations in SSR repeat length. To this end, we employed multiple strategies to minimize bias and enhance analytical accuracy. (i) Low-quality reads (quality score < 20) were removed from the resequencing datasets. (ii) The uniqueness and specificity of SSRs were assessed using e-PCR (a computational simulation of PCR amplification), thereby reducing misalignment errors. (iii) Single-locus SSR markers were chosen to avoid genotype scoring errors arising from overlapping loci and uncertain allelism [55]. (iv) Alignment parameters were optimized by allowing one mismatch, balancing alignment stringency with tolerance for SNPs and other genomic variations. (v) Multiple germplasm accessions from diverse ecological regions were included to facilitate the detection of polymorphic SSR loci, owing to their high genetic diversity and unique alleles.

In this study, we identified 121,544 unique SSRs that were polymorphic across 15 genotypes. These DNA polymorphisms, which reflect underlying genetic variation, form the basis for developing molecular markers. PIC values are an important indicator of molecular marker informativeness, and markers with PIC values ≥ 0.5 are generally considered highly informative [56]. For the common SSR loci detected across ten or more genomes, PIC values ranged from 0.13 to 0.92, with an average of 0.59. We successfully designed 10,364 SSR markers from the flanking sequences of common SSR loci with PIC values ≥ 0.5 and major allele size differences ≥ 3 bp. Experimental validation in a panel of 45 *D. officinale* germplasm accessions showed that the majority (90%) of the newly developed SSRs exhibited moderate to high polymorphism, with PIC values ranging from 0.31 to 0.74. These results suggest that reference genome assemblies and resequencing data offer an efficient means of characterizing genome-wide polymorphic SSR markers. Discrepancies in allele number and PIC values between *in silico* analysis and experimental validation may result from the different resolution capabilities of e-PCR and gel electrophoresis. Alternatively, these discrepancies may be attributable to genuine genetic differences between the *in silico* panel and the validation population. In addition, sequencing errors and the bioinformatic algorithms employed may have contributed to inaccuracies in estimating polymorphism levels from the sequencing data. A key advantage of these newly developed genomic SSR markers is their high polymorphism, as confirmed through experimental validation. Furthermore, these markers can be conveniently genotyped using gel electrophoresis. The abundant allelic variation they reveal enables precise discrimination among diverse germplasm accessions, facilitating the assessment of genetic diversity and population structure. In addition, a high-density set of genomic SSR markers provides abundant genetic information and genome-wide coverage, enabling high-resolution genetic mapping, precise QTL analysis, rapid gene cloning, detailed evaluation of genetic diversity, and efficient marker-assisted breeding. Collectively, these features make them a powerful resource for both fundamental research and practical breeding applications in *D. officinale* and related species.

## 4. Materials and Methods

### 4.1. Plant Materials and Genome Sequencing

Whole-genome resequencing (WGRS) was performed on 14 *D. officinale* germplasm accessions collected from diverse regions in China (Table 4). Genomic DNA was extracted from young leaves using a modified cetyltrimethylammonium bromide (CTAB) method. DNA quality and concentration were determined using a Qubit 2.0 Fluorometer (Thermo Fisher Scientific, Waltham, MA, USA). Paired-end sequencing libraries were constructed for each sample following the Illumina standard protocol. The libraries were sequenced on an Illumina HiSeq 2500 platform (Illumina, San Diego, CA, USA) to generate 150-bp paired-end reads. Each sample was sequenced to a minimum depth of 8.69×, with a genome coverage of at least 93.20%. The reference genome sequence of *D. officinale* “Niu2020” was retrieved from the NCBI database. Using the original annotation file of the “Niu2020” reference genome, we extracted the genomic coordinates of 2 kb upstream of the transcription start sites (TSS_up_2Kb), 5′ and 3′ untranslated regions (UTRs), protein-coding sequences (CDSs), introns, 1 kb downstream of the transcription end sites (TES_down_1kb), and intergenic regions. To validate the developed SSR markers, PCR amplification was conducted on 45 *D. officinale* germplasm accessions originating from diverse geographic regions (Table S8).

### 4.2. SSR Screening and Identification of Unique Loci

SSR loci were identified using the MIcroSAtellite identification tool (MISA; http://pgrc.ipk-gatersleben.de/misa/, accessed on 1 November 2022). Only perfect SSRs with at least 10, 7, 6, 5, 4, and 4 repeat units for mono-, di-, tri-, tetra-, penta-, and hexa-nucleotide motifs, respectively, were considered. SSRs separated by less than 100 bp were classified as compound SSRs. For each SSR locus, 20-bp flanking sequences starting 5 bp upstream and downstream were extracted using a custom Perl script. The upstream sequences were used as forward primers, whereas the downstream sequences were reverse-complemented to serve as reverse primers for e-PCR. The primer sequences were aligned to the *D. officinale* reference genome “Niu2020” using Bowtie [57], allowing up to three mismatches. SSR loci with primer pairs that aligned to multiple genomic positions were excluded to ensure the amplification of unique, locus-specific products.

### 4.3. SSR Variation in D. officinale Genomes

Raw sequencing reads were quality-filtered using the NGSQC Toolkit v2.3.3 [58]. De novo assembly was performed with ABySS v1.5.2 [59] using a k-mer size of 25. Unique SSR primer pairs were aligned to filtered reads from 14 *D. officinale* germplasm accessions using Bowtie, allowing up to one mismatch. The amplicon length generated by each e-PCR primer pair was extracted using a custom Perl script. To ensure locus specificity, SSR loci were excluded if *in silico* amplification produced multiple amplicons in any of the 14 accessions. For each SSR locus with length information available in at least ten accessions, allelic diversity was calculated based on the polymorphic information content (PIC), as described by Anderson et al. [60].

$$\mathrm{PIC}i=1-\sum_{j=1}^{n}p_{ij}^{2}$$

where *p_ij_* is the frequency of the *j*th pattern for the *i*th marker.

### 4.4. Primer Design and Experimental Validation of Polymorphic SSRs

Primers were designed for unique SSR loci with PIC values ≥ 0.5 and major allele size differences ≥ 3 bp. For each selected SSR locus, the SSR motif and 100-bp flanking sequences on both sides were used for automated primer design using Primer3. Primers were designed according to the following criteria: a length of 17–25 bp, an annealing temperature of 55–65 °C, a GC content of 40–60%, and an expected PCR product size of 70–300 bp. To further evaluate the utility of the identified SSR markers, 20 primer pairs were randomly selected for experimental validation. These primers were validated using 45 *D. officinale* germplasm accessions collected from diverse regions across China. Genomic DNA was extracted from young leaves as described above. PCR amplification was performed in a 25 µL reaction mixture containing 100 ng of genomic DNA, 12.5 µL of 2 × 3G Taq Master Mix for PAGE (Vazyme, Nanjing, China), 100 nM of each SSR primer, and ddH_2_O to final volume. The PCR cycling conditions were as follows: initial denaturation at 94 °C for 5 min; 35 cycles of denaturation at 94 °C for 30 s, annealing at 57 °C for 30 s, and extension at 72 °C for 40 s; and a final extension at 72 °C for 8 min. PCR products were resolved on a 6% denaturing polyacrylamide gel and visualized by silver staining. The number of alleles per locus was scored, and the PIC value for each marker was calculated as described above.

## 5. Conclusions

This study presents a genome-wide characterization of SSR loci in *D. officinale* and reports the development of polymorphic genomic SSR markers through the integration of the reference genome and resequencing data. In total, we identified 388,553 unique SSR loci and developed 10,364 primer pairs to target highly polymorphic sites. Experimental validation demonstrated a high success rate, with 90% of the selected primers successfully amplifying polymorphic loci. This comprehensive set of genome-wide SSR markers substantially enriches the molecular marker resources of *D. officinale* and is expected to facilitate diverse applications, including genetic diversity assessment, cultivar identification, genetic linkage and QTL mapping, gene cloning, and marker-assisted selection. Overall, these resources provide robust molecular tools to support both fundamental and applied research in *D. officinale* and related species.

## Figures and Tables

**Table 1:** Distribution of SSR types in the *Dendrobium officinale* genome.

Types	Repeat Units	Overall SSRs ^a^				Unique SSRs ^b^			
		Count	Length (bp)	GC (%)	Rate ^c^ (%)	Count	Length (bp)	GC (%)	Rate ^d^ (%)
Simple SSRs	MNRs	329,390	12.64	11.62	66.57	259,178	12.70	11.74	66.70
	DNRs	91,524	34.14	17.17	18.50	71,921	35.96	15.65	18.51
	TNRs	37,451	19.93	22.28	7.57	28,766	20.18	20.57	7.40
	TTRs	2235	21.51	11.42	0.45	1861	21.43	9.42	0.48
	PNRs	448	26.30	18.60	0.09	326	26.34	18.52	0.08
	HNRs	222	32.48	44.05	0.04	165	32.50	45.37	0.04
	Total	461,270	17.57	14.78	93.23	362,217	17.98	14.10	93.22
Compound SSRs ^e^	--	31,384	96.40	23.50	6.34	24,590	63.03	22.02	6.33
Compound* SSRs ^f^	--	2129	63.26	22.56	0.43	1746	60.16	23.66	0.45
Total	--	494,783	22.76	17.22	100.00	388,553	21.02	15.73	100.00

Note: ^a^ Overall SSRs were identified across all 19 chromosomes; ^b^ Unique SSRs were defined as those with unique flanking sequences in the genome; ^c^ The percentages of SSRs with different repeat units were calculated relative to the overall SSRs; ^d^ The percentages of unique SSRs with different repeat units were calculated relative to the unique SSRs; ^e^ Compound SSRs: composed of two or more contiguous simple SSR motifs without intervening sequences; ^f^ Compound* SSRs: composed of two or more simple SSR motifs interrupted or separated by one or more nucleotides.

**Table 2:** Frequency and distribution of SSRs in different genomic regions.

Genome Regions	Overall SSRs				Unique SSRs				
	Count	Interval ^a^ (Kbp)	Length (bp)	GC (%)	Count	Interval ^a^ (Kbp)	Length (bp)	GC (%)	Rate ^b^ (%)
TSS_up_2Kb	32,278	0.63	20.80	12.94	27,848	0.54	20.82	12.25	86.28
5’UTR	1320	0.69	18.79	36.09	1132	0.59	17.07	19.17	85.76
3’UTR	998	0.53	15.14	11.54	944	0.50	15.23	11.16	94.59
CDS	1299	0.05	17.42	54.39	1205	0.04	17.26	54.53	92.76
Intron	177,177	0.43	22.27	17.94	140,309	0.34	14.12	14.44	79.19
TES_down_1kb	17,603	0.69	19.11	10.49	15,583	0.61	18.74	9.85	88.52
Intergenic	264,108	0.42	23.60	18.76	201,532	0.32	14.68	14.77	76.31
Total	494,783	0.43	22.76	17.99	388,553	0.34	20.86	16.34	78.53

Note: ^a^ Interval was calculated as the number of SSRs per Kbp; ^b^ The percentage of unique SSRs was calculated relative to the overall SSRs in each genomic region.

**Table 3:** Characteristics of 20 SSR markers used for experimental validation.

Primer Name	SSR Type	e-PCR		PCR Validation		PCR-Based Primer		
		Allele Number	PIC	Allele Number	PIC	Forward	Reverse	Product Size (bp)
Chr1-459196	(T)10	3	0.60	4	0.54	CATTCACTAACGACCACATTTGA	CCGATAGGCCTAATCCTGTTC	103
Chr1-463581	(A)16	4	0.65	3	0.59	AATTGCTAACCAGGCCATCA	TCTTCGATCCCTTGACTGCT	136
Chr1-59422185	(TGT)8	2	0.50	6	0.51	AAGGATTCATGTAGCCGACC	GGAACGAAAATATTATGTGCCAA	112
Chr1-508186	(TTAT)5	4	0.56	4	0.31	TTTCTAGTGTCGCTCTGAAATG	CCTGATTCACGCTCACATTG	105
Chr2-4294007	(T)10	4	0.68	4	0.69	CTAACCACACCCATCCCAAC	CGGCGGTAAGCCTAACTTTT	135
Chr2-47634991	(ATTT)5	2	0.50	1	0.00	CAAAGCTCATGAAAGCTTCAAC	TTCAAAGAAAAATACAATCCCCA	165
Chr2-5701487	(TCT)5	3	0.62	2	0.43	TTGCTCATAAACACACACTCTCTC	CAACAGGAAGGACTAGAGGATCA	104
Chr2-78041173	(CAAAA)5	2	0.50	2	0.39	AAAGAGAACCAATCTACTGAACGG	GCATCTGCCTTTTGAATCTTCT	129
Chr3-7889288	(TA)6	2	0.50	/	/	AAGATGGTACAGAAAGCAGAGACA	TTTTTCATCTTTCTTTGTTTGAGC	90
Chr3-3538709	(TGT)5	3	0.50	3	0.54	CCACGTAGTGGGATAAAGGC	GTATCAGGGGGCATTGATGT	116
Chr3-5676585	(ATT)6	3	0.58	3	0.58	CCAGTTTAATTTTTGGGATTCA	TGACCATAAACGTTGCCTGA	110
Chr3-16813768	(AAT)10	4	0.66	4	0.69	GGGCTAATGCAAAATGTAGTTG	TGATTCGTTTTATGTTTATTTGGG	130
Chr4-23399662	(TTTA)6	2	0.50	4	0.52	CCCAATAATGAATGCAGCTCT	CAAAAATGATTGAAAACAACTCCA	160
Chr4-41229100	(CCCT)5	3	0.60	4	0.69	GGGTACCAACCGGCCTAT	AAGAGAGGGAGAGGGAGAGG	88
Chr4-55668381	(GGGTG)6	4	0.71	5	0.66	TCACGGCTAGCTTCTTGAGAG	GCCAAATGTCCTCCCTCTTT	100
Chr4-7402326	(TGT)8	5	0.59	5	0.53	TTCATGTAGCCGACCCTATG	CAGGTCACCAAAAGTTATTTGC	118
Chr5-16940219	(TGT)5	2	0.50	3	0.49	TGGATTCATATAGTCGACCCAA	AAGCTGGCCATAAAGAGGGT	83
Chr5-2512207	(AAGC)5	3	0.57	3	0.56	CTAAATTAGTTTGGCAGCACAGT	TCATCCTTCTGCAGTGAGAAAA	109
Chr5-2550036	(GAT)8	4	0.63	4	0.67	TTCAGAGTATTCATGGGATTTCA	AATATCACCATTCCAAAGAGTTTC	100
Chr5-4384422	(AT)9	5	0.68	4	0.74	TTCAACATTTGACTCGACTGTACT	AAACAAAGTGAAAGGAAGGCT	121

**Table 4:** Geographic origins of 14 *Dendrobium officinale* germplasm accessions subjected to whole-genome resequencing.

Sample	Accession Number	Geographic Origin
1	3	Huangshan, Anhui
2	A82	Pucheng, Fujian
3	A52	Taining, Fujian
4	A44	Wuyishan, Fujian
5	GHY	Heyuan, Guangdong
6	A17	Renhua, Guangdong
7	A34	Rong, Guangxi
8	A39	Pingjiang, Hunan
9	A31	Guangfeng, Jiangxi
10	A32	Longnan, Jiangxi
11	HY	Hanyuan, Sichuan
12	LS	Liandu, Zhejiang
13	XJ1	Xianju, Zhejiang
14	A23	Xingguo, Jiangxi

## Data Availability

All data are available in the article and its Supplementary Materials. Any further inquiries can be directed to the corresponding authors.

## Supplementary Materials

The following supporting information can be downloaded at: https://www.mdpi.com/article/10.3390/plants14233589/s1, Table S1: SSRs identified in the *Dendrobium officinale* Niu2020 genome; Table S2: The proportion of SSR motifs in the *Dendrobium officinale* Niu2020 genome; Table S3: Summary of whole-genome resequencing data for 14 *Dendrobium officinale* genotypes; Table S4: The e-PCR products of unique SSRs in *Dendrobium officinale* germplasm accessions; Table S5: The distribution of SSRs in the *Dendrobium officinale* genome; Table S6: PIC values of common SSR loci; Table S7: Details of polymorphic SSR markers developed in this study; Table S8: Information on 45 *Dendrobium officinale* germplasm accessions for PCR validation.
